# Probability distribution of copy number alterations along the genome: an algorithm to distinguish different tumour profiles

**DOI:** 10.1038/s41598-020-71859-1

**Published:** 2020-09-10

**Authors:** Luísa Esteves, Francisco Caramelo, Ilda Patrícia Ribeiro, Isabel M. Carreira, Joana Barbosa de Melo

**Affiliations:** 1 Cytogenetics and Genomics Laboratory, Faculty of Medicine, University of Coimbra, Polo Ciências da Saúde, 3000-354 Coimbra, Portugal; 2 Laboratory of Biostatistics and Medical Informatics, IBILI-Faculty of Medicine, University of Coimbra, 3000-354 Coimbra, Portugal; 3 iCBR-CIMAGO-Center of Investigation on Environment, Genetics and Oncobiology-Faculty of Medicine, University of Coimbra, Coimbra, Portugal

**Keywords:** Cancer genomics, Scientific data

## Abstract

Copy number alterations (CNAs) comprise deletions or amplifications of fragments of genomic material that are particularly common in cancer and play a major contribution in its development and progression. High resolution microarray-based genome-wide technologies have been widely used to detect CNAs, generating complex datasets that require further steps to allow for the determination of meaningful results. In this work, we propose a methodology to determine common regions of CNAs from these datasets, that in turn are used to infer the probability distribution of disease profiles in the population. This methodology was validated using simulated data and assessed using real data from Head and Neck Squamous Cell Carcinoma and Lung Adenocarcinoma, from the TCGA platform. Probability distribution profiles were produced allowing for the distinction between different phenotypic groups established within that cohort. This method may be used to distinguish between groups in the diseased population, within well-established degrees of confidence. The application of such methods may be of greater value in the clinical context both as a diagnostic or prognostic tool and, even as a useful way for helping to establish the most adequate treatment and care plans.

## Introduction

DNA copy number is naturally variable and these copy number variations (CNVs) are commonly observed in the germline and are thus inherited^1^. Copy number alterations (CNAs) comprise deletions or amplifications of genomic material fragments, with a size as low as a few kilobases up to entire chromosomes that are often longer than CNVs^1,2^. Somatically acquired copy number alterations (SCNAs) are particularly common in cancer and play a major contribution in its development^3–6^. Genes are affected at a functional level by this kind of genetic aberration with impact in the phenotype^7^. However, as cancer progresses, genomic instability often increases originating multiple apparently random alterations. In addition to cancer, CNAs have also been linked to other diseases such as autoimmune diseases, Alzheimer’s and Parkinson’s Disease^8,9^. One of the challenges in genome-wide analysis of SCNAs is to distinguish between the alterations that are critical in terms of disease origin and progression—driver alterations—and those that occur as a consequence of genomic instability during progression—passenger alterations^3,4,10^. Both these two types of alterations need to be further studied and characterized in order to understand the aetiology of the disease as well as their potential role in new targeted therapies.

Currently, microarray-based genome-wide technologies such as array-based comparative genomic hybridization (aCGH) and single nucleotide polymorphisms (SNP) arrays have been widely used in what comes to CNA detection, at progressively higher resolutions^11–13^. These technologies allow measuring copy number for hundreds of thousands of probes along the genome and their potential has been exploited by the growing number of readily available large datasets from cohort studies for various types of pathologies, in which cancer has a dominant presence. However, these complex datasets have some difficult processing and statistical challenges^14^. Frequently, the available data are segmented tumour samples where individual genomic regions-sequences of altered probes—are “called” as gains or losses, based on their amplitudes by choosing a significant statistics or threshold, reducing measurement noise and resulting on the segments boundaries (breakpoints). These regions can then be used to define recurrent or common CNAs—sets of contiguous probes that have a high enough probability to be altered in at least some tumour samples of the cohort. These common frequently occurring regions of alteration are determinant to the detection of driver mutations important to the progression of the disease. This assumption is based on the fact that CNAs functionally associated to the disease will be present in a considerable amount of the analysed genomes as opposed to random somatic mutations that are subject-specific and as such will be present in a restricted number of subjects^14^. Several methods have been employed to determine these commons CNAs^10^, amongst those Genomic Identification of Significant Targets in Cancer (GISTIC) seems to be the most used^15,16^.

In this work, however, the main focus is not on single driver alterations but on the complete set of copy number alterations present in a cohort, creating a disease profile across the sample. In this context, driver alterations can still be identified as those with a higher probability of alteration but are not the main target. This approach, nevertheless, does not fit in with most methods that determine common copy number alterations with the purpose of identifying driver alterations.

Here we present a method for the determination of recurrent CNAs, based on segmented individual genomic profiles with the ultimate goal of determining the underlying probability distribution of these CNAs in the disease under study, establishing disease profiles.

## Methods

### Introduction and rationale

#### Context of the problem

Causality in epidemiological studies is at least very difficult to prove. This is due to the fact that there are several non-controlled conditions and to the natural biological variation. Despite these constraints, a strategy to overcome this issue was devised, which has allowed to improve medicine and health care. Risk is the main idea behind this strategy and the determination of the risk given an exposition factor allows better managing of health care. Although risk is a powerful concept it is based on the assumption that the relationship between the disease and factors are stochastic. Genomic studies share the same model, in particular association studies between genetic variations and disease assume the following rationale:

1. There is natural genetic variation, which is not linked to disease;
2. There is specific genetic variation that is strongly associated to disease;
3. Genetic variation is stochastic and is an unknown function.

Points 1 and 2 mean that for a particular subject carrying a disease his genomic profile would present genetic variation that only in part could be linked to the disease. The major problem is that it is impossible to distinguish the part linked to the disease and the part that is natural random genetic variation. Scaling up the problem for a set of subjects, the two components of the genetic variation could in theory be identifiable since the common genetic variation would be linked to the disease. However, even this component has a random structure making the identification very difficult.

Hence, and since both the natural and the specific components of genetic variation are stochastic, the particular manifestation of this variation in each person is different. If the phenomenon was deterministic, the problem of knowing the genetic variation associated to one specific disease would be straightforward, or at least simpler, because in that case the variation would be equal in each diseased person. However, as stated before, the phenomenon is better modelled if considered stochastic and, thus, may be modelled by an unknown probabilistic function. Given that, the problem is to find the probabilistic function associated to the specific variation component.

#### Direct problem: from the population to a sample of subjects

The concept we propose is that the real genetic signature of a disease is given by a mathematical function describing the probability of different genetic regions being altered in the DNA. In this context, a region is a contiguous set of DNA base pairs that could be defined by a fixed or a variable number of base pairs, but once defined the size of the different regions one should consider them fixed in order to allow comparisons between subjects.

To better understand the problem and the notion of disease signature in this situation, we shall consider the following synthetic very simple example. This example concerns the direct problem, which means that knowing the function that defines the signature of the disease a set of subjects can be simulated. In practice, we are interested in solving the inverse problem which is, given a set of subjects to find the function that defines the signature of the disease. It is easier, however, to firstly understand the direct problem and then tackle the inverse one.

**Figure 1:**
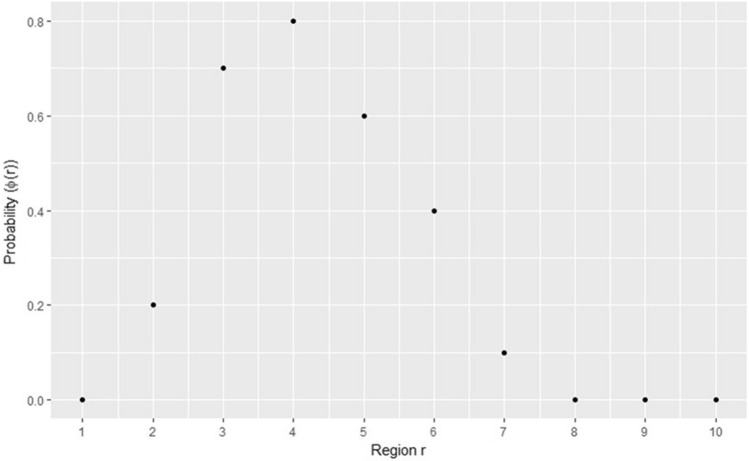
Probability of alteration for each region r. In the y axis is represented the probability of alteration of each region that is represented in the x axis.

For the sake of simplicity, but with no loss of generality, we will assume only 10 genomic regions of the same length and each one having an unknown number of pair base. The function that defines the disease signature, or simply the signature, could then be defined by a discrete function of probability, $\Phi \left( r \right)$, given by the following vector of values and the corresponding chart (Fig. 1).

$$\Phi \left( r \right) = \left\{ {0.0, 0.2,0.7,0.8,0.6,0.4,0.1,0.0,0.0,0.0} \right\}.$$

In this simple example, for each genomic region, $r$, a Bernoulli experience is assumed with probability given by the function, $\Phi \left( r \right)$. A given patient in this context is defined by 10 regions that can either be normal, 0, or altered, 1. The number of regions is arbitrary and continuous functions and more complex outcomes, namely different degree of alteration, might be devised as signature functions; however for keeping the explanation simple we are only considering two states, thus the Bernoulli experience was considered. In this framework a patient having this disease is then modelled as a random draw with a probability given by the function, $\Phi \left( r \right)$. From a statistical point of view, the function $\Phi \left( r \right)$ defines the population, which is considered as the set of all the subjects that have the same features. Considering now five random draws with probability given by $\Phi \left( r \right)$ we can say that these draws represent five different patients, $f_{1}$ to $f_{5}$, that have this disease. Hence, the patients may be represented by a vector of values, where 0 means that that region is not altered and 1 means otherwise:

$$f_{1} = \left\{ {0, 1,1,1,0,0,0,0,0,0} \right\},$$

$$f_{2} = \left\{ {0, 0,1,1,0,0,0,0,0,0} \right\},$$

$$f_{3} = \left\{ {0, 0,1,1,1,0,0,0,0,0} \right\},$$

$$f_{4} = \left\{ {0, 1,0,1,1,0,0,0,0,0} \right\},$$

$$f_{5} = \left\{ {0, 1,0,1,1,1,0,0,0,0} \right\}.$$

In this synthetic case, the five patients present different genomic profiles but we are assuming that they have the same disease. This is a very simple example aiming to illustrate the main idea; nonetheless, it can be perceived that the five patients have similar profiles, which is expected since they come from the same random function.

#### Inverse problem: from the subjects to definition of the population

As mentioned before, the question in practice is how to solve the inverse problem: knowing the patients’ genomic profiles how can we obtain the function that defines the genomic signature of the disease. The genomic signature, or more redundantly the true genomic signature is in fact an unknown function, but it can be estimated using large samples of patients and the larger the sample the better the estimation to the true signature. In the example presented, one may state that region 1 has very low probability ($P\left( 1 \right) \approx 0$) of being altered since none of the patients have alterations in this region. In contrast, region 4 is highly likely to be altered because all the patients have alterations in that region. Taken this into account, the question is how to compute the probability that can explain this particular sample.

The way we see the problem is such that it has two different components. On one hand, the number and the size of the regions have to be defined because the signature function will be defined based on these parameters. In addition, it is necessary to consider if the regions have hard boundaries or fuzzy limits. In the first case, a piecewise-defined function would be more suitable and for the latter a continuous function may be more appropriate. On the other hand, it is necessary to define the outcome of the function, which would lead to different types of functions. The outcome could be simple binary, as presented in the example, or could be multiclass or even continuous. The two types of sub problems present different challenges and could be easier or harder to address according to their definition. In the present work, we present a solution for the case where the regions have hard boundaries and the function has a binary outcome. The algorithms are detailed in the following sections.

### Partition algorithms and determination of the probability of alteration

In order to estimate the signature function, the definition of the regions has to be settled, because the function itself is based on the regions and the genetic profiles of all patients have to be described in the same representation space. To determine which regions are most commonly altered in a set of patients, we have to make sense of the vast array of data contained in each patient’s genetic profile and represent only the relevant information.

The genome-wide techniques used for the detection of CNAS are very sensitive and have a high resolution generating highly complex datasets. Nevertheless, they are also invaluable to the identification of CNAs involved in the development and progression of the disease being studied. As such, a method for data compression is necessary in order to find common CNAs amongst the alterations present in the cohort. In this scenario, we consider that all samples in the study are homogenous as we want to focus on evident patterns in the data, independently of inter or intra-tumour heterogeneity within the sample, due to the presence of disease subtypes or different tissues of tumour origin.

The segments, defined as sequences of probes with the same copy number, are determined by a segmentation algorithm prior to the implementation of our method, meaning that the successive probes are correctly assigned to the same copy number. We are aware that data will have errors due to the limitations of the technique and that the spatial resolution is particularly affected, which means that some pair bases will be wrongly identified as being altered probably due to aliasing of the signal from highly altered DNA portions. This effect will be present in all the genetic profile and it is impossible to distinguish the genuine altered regions from the wrongly classified as such. However, for a given set of profiles it is less likely that these artefacts would be present for all subjects at the same position.

Furthermore, there is high variation in the CNAs detected even within the subjects that have the same disease, however some CNAs have a higher probability of being altered than others.

**Figure 2:**
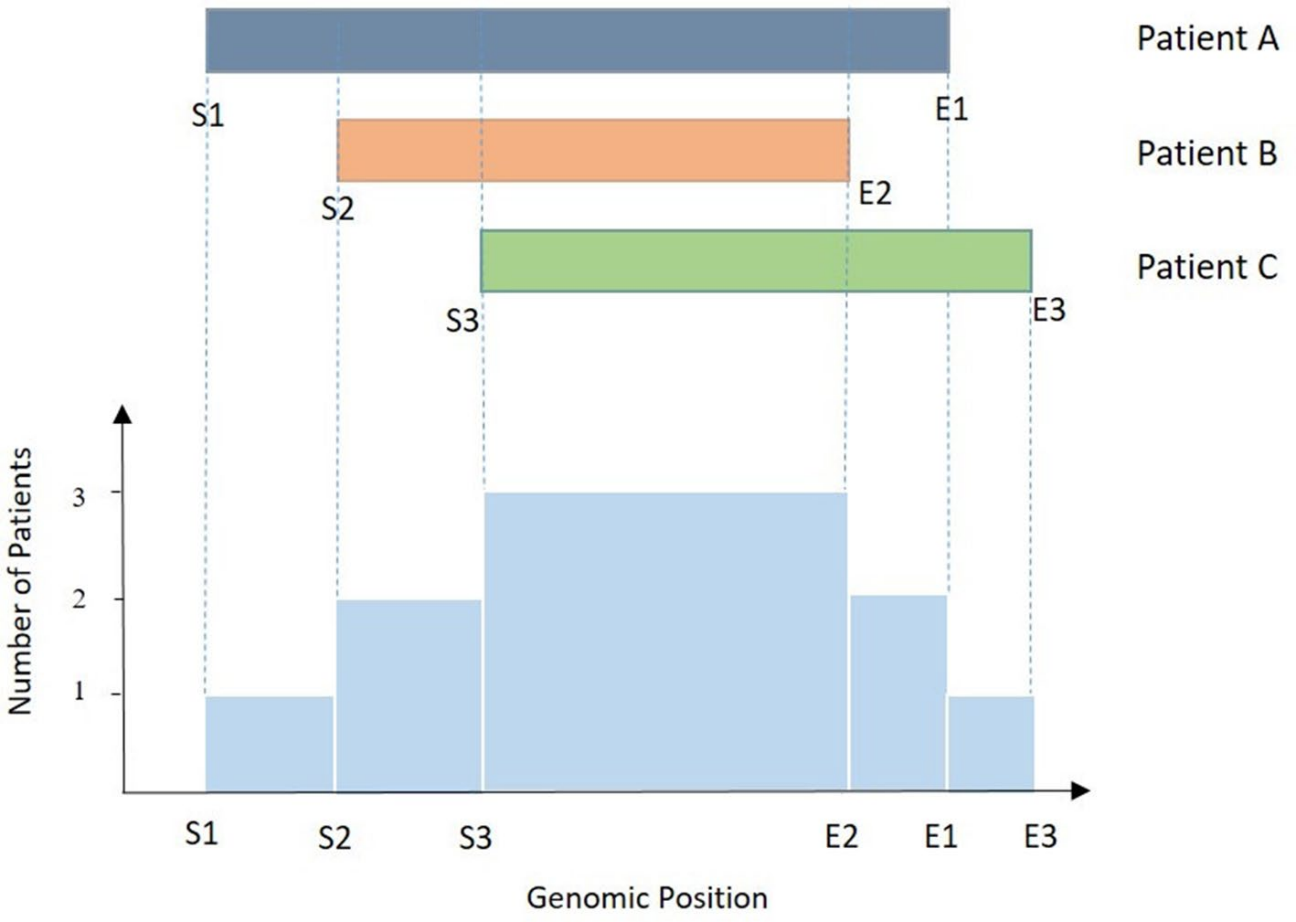
Schematic representation for the process to determine the common regions of alteration by Algorithm 1. The grey, orange and green bars represent altered regions in each one of the patients (**A**–**C**) and are limited by their respective Starts (S1–S3) and Ends (E1–E3). The regions resulting from Algorithm 1 are represented in light blue, with the aligned genomic positions (Starts and Ends) defining their limits. The height of the light blue bars represents the number of patients that present an alteration in that given region.

Our algorithm partitions a dataset containing aCGH or SNP data from several subjects that have been subject to a segmentation algorithm. It recovers the CNAs by chromosome with their respective probability of alteration. To achieve this, we consider the set of genomic profiles within the dataset, in a given chromosome, and aggregate them in a single profile containing the overlapping regions and their breakpoints. This procedure generates a more structured dataset and reduces the complexity of the data. The algorithm used to find the common regions of alteration in a given chromosome (Algorithm 1) takes as input two arrays: one defining the starts of all the regions altered in one set of subjects, and the other containing the ends of the same regions. An identifier number is associated to each breakpoint: 1 identifies the start and 0 identifies the end of a region. The goal of this algorithm is to find the common regions of alteration by merging both arrays into a single one and then sorting it in ascending order. This step allows to easily determinate the adjacent breakpoints regardless of their identifiers, and simplifies the parsing of the data. The common regions are calculated taking neighbouring breakpoints (starts or ends) that do not fall in the same loci, using them to define the limits of the new regions (Fig. 2). The second goal of the algorithm is to track the number of regions of alteration that intercept each one of the calculated common regions, that is, to calculate the number of subjects with an alteration in a given common region. This is achieved via a counter variable that uses the identifiers to determine when a breakpoint is a start or an end, adding one subject for each start and subtracting one for the corresponding end.

Algorithm 1 can be used either taking into account amplifications and deletions or just alterations in general without distinction. Besides finding the common regions of alteration, it determines the number of patients with alteration in those regions and also computes a matrix containing each subject’s alterations as it relates to the common regions.

The size of regions tends to be different as a natural result of the application of the algorithm (Fig. 2).

Assuming that the common regions are independent from each other, the estimated probability of alteration, $$\hat{\Phi }\left( r \right),$$ of a chromosomic region, $$r$$, is directly given by the quotient between the number of occurrences in that region ($$n_{occurences\,\,r} )$$ and the total number of subjects in the cohort ($$N_{subjects} )$$.

1 $$\hat{\Phi }\left( r \right) = \frac{{n_{occurrences} \left( r \right)}}{{N_{subjects} }}.$$

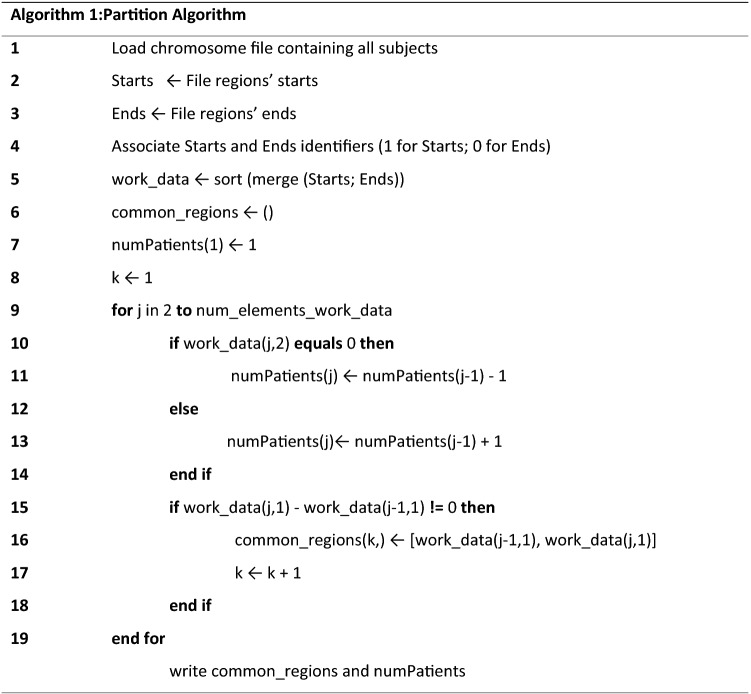


The partition algorithm was included in a Matlab (Mathworks™) standalone program that allows the user to compute the common regions and to generate the corresponding plots.

### Testing of the partition algorithm in simulated data

To debug and validate the partition algorithm regarding its ability to determine the common regions of alteration accurately, we implemented a routine that generates simulated common regions of alteration across a given set of samples (Algorithm 2). This algorithm takes three parameters: the number of regions, the number of patients and one generator function. A region in the algorithm is perceived as a block that comprehends a number of base pairs and, each of these regions admits only two states: altered or normal or three states: gain, loss or normal. When the user defines the number of regions he is defining the length of the simulated DNA code that is under analysis. Another parameter is the number of subjects, which defines the size of the sample. The main operation within the algorithm is to randomly label each region, as being altered or normal and, in order to achieve this, it uses one generator function that defines the probability of each region being altered. The generator function is defined as a vector where each element represents the probability of a binary event takes place. The number of the vector elements is equal to the number of regions as depicted in Fig. 1.
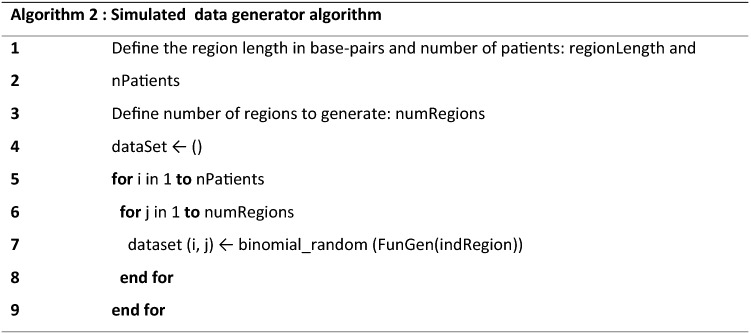


A simplified example of a generated dataset is presented in Table 1.

**Table 1:** Example of a simulated dataset (from Algorithm 2).

	R1	R2	R3	R4	R5
S1	0	0	0	0	1
S2	0	0	0	1	1
S3	0	1	1	1	0
S4	0	0	0	1	1
S5	0	0	1	0	1

Alteration status (0 for not altered and 1 for altered) for the simulated common regions R1–R6 in sample with 5 subjects, from S1 to S5.


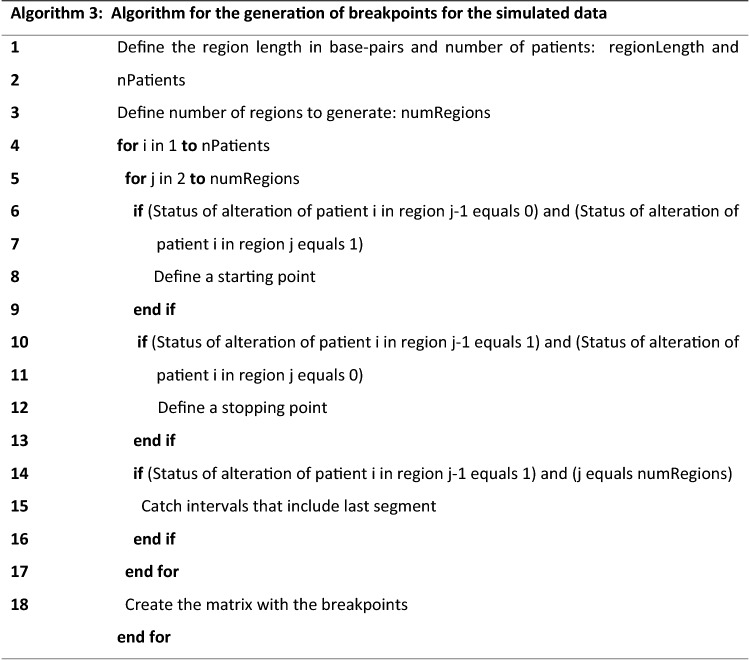


The results from Algorithm 2, as exemplified in Table 1, are then used as input in Algorithm 3.

The main purpose of this algorithm is to construct a simulated dataset equivalent to the separate altered regions in each subject, with starts and ends similar to what happens with real data. These data can then be used as an input for Algorithm 1. The result of using Algorithm 3 in Table 1 is shown in Table 2.

**Table 2:** Summary of the result obtained by applying Algorithm 3 to the regions defined in Table 1.

Sample	Start	End	Regions
S1	4,000	5,000	R5
S2	3,000	4,000	R4
	4,000	5,000	R5
S3	1,000	2,000	R2
	2,000	3,000	R3
	3,000	4,000	R4
S4	3,000	4,000	R4
	4,000	5,000	R5
S5	2000	3,000	R3
	4,000	5,000	R5

Regions in Table 1 assume a set size of 1,000 base pairs.

### Determination of the error for the generator function in simulated data

The real generator function $\Phi \left( r \right)$ in the simulated data is known since it is given at the start of the routine (Algorithm 2) and is used to create the datasets. However, after applying the partition algorithm, a new generator function is computed – the estimated generator function $\hat{\Phi }\left( r \right)$. We must evaluate the error between $\Phi \left( r \right)$ and $\hat{\Phi }\left( r \right)$, which is defined as follows by Eq. (2):

2 $$\varepsilon = \mathop \sum \limits_{r,i}^{R,N} \frac{{\left( {\Phi \left( r \right) - \hat{\Phi }\left( r \right)} \right)_{i}^{2} }}{{N_{subjects} }}.$$

Using the same initial generator function, we simulated progressively larger sets of samples and used them to evaluate the error $\varepsilon$.

We also evaluated the percentage error between $\Phi \left( r \right)$ and $\hat{\Phi }\left( r \right)$ for the same simulated datasets defined by Eq. (3):

3 $$\varepsilon_{p} = \mathop \sum \limits_{r,i}^{R,N} \frac{{\left( {\Phi \left( r \right) - \hat{\Phi }\left( r \right)} \right)_{i}^{2} }}{{\Phi \left( r \right)_{i} }}.$$

We evaluated forty simulated datasets, correspondent to four different generator functions, with lengths varying from 100 to 1,000 subjects, with a spacing of 100 subjects.

### Real data

#### Head and neck squamous cell carcinoma cohort analysis

In order to explore the properties of our algorithm in a dataset containing real data we used a cohort of Head and Neck Squamous Cell Carcinoma (HNSCC) containing 522 samples of tumour tissue, downloaded from the The Cancer Genome Atlas (TCGA) Database using the Broad Institute's Firehose Pipeline. TCGA has been widely used in individual cancer type studies as well as in pan-cancer analysis^17–20^.

DNA was hybridized in an Affymetrix Genome-Wide Human SNP Array 6.0 platform. The downloaded data was already mapped to the hg19 reference genome and segmented by the Circular Binary Segmentation algorithm, and all known copy number variation probes were already removed.

HNSCC is a very heterogeneous disease that arises in the upper aerodigestive tract and is the sixth most incident cancer worldwide with a 5-year survival rate of about 50%. Normally these carcinomas are diagnosed at later clinicopathological stages. The most important risk factors associated with HNSCC are tobacco use and alcohol consumption, that seem to have a synergistic relationship. A subgroup of HNSCCs especially prevalent in the oropharynx caused by high-risk human papillomavirus (HPV) has also been identified as a completely different clinicopathological and molecular entity from HPV-negative tumours^21,22^.

Firstly, data was separated by chromosome and was filtered by number of probes and by log_2_ segment mean. Then the previously described partition algorithm (Algorithm 1) was applied and the common regions of alteration were computed.

The samples were separated in two groups: HPV-positive (n = 99) and HPV-negative (n = 423) and for each of the groups the probability of alteration for every common region was determined. Two plots depicting amplified and deleted regions for both HPV-negative and HPV-positive groups were created. Besides, subsampling was performed to analyse the robustness of the algorithm and the impact of having smaller sample sizes. Three sample sizes were tested; 50, 100 and 200 subjects were randomly drawn from the original dataset while maintaining the HPV proportion.

### Lung adenocarcinoma cohort analysis

A disease profile for a different cancer, lung adenocarcinoma, was also determined based on the proposed method. Adenocarcinoma is the most common type of lung cancer, either in smoking and non-smoking men and women, despite their age (Zappa and Mousa, 2016). The data from this cohort contains 516 patients and was also extracted from the TCGA Database and pre-processed in a similar way to the HNSCC data.

## Results

### Determination of the error for the generator function in simulated data

We verified that the algorithm performed well for all the forty simulated datasets, retrieving correctly the common regions of alteration. The method employed is based on intercepting regions coming from the data and thus this was the expected outcome.

**Figure 3:**
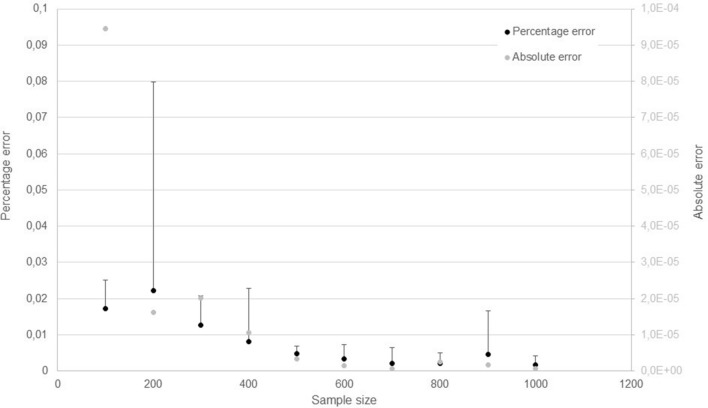
Representation of the average calculated errors between the four real and estimated generator functions in simulated data, as a function of the number of patients in the cohort (between 100 and 1,000 subjects, with an interval of 100), along with corresponding standard deviation bars defined as two standard deviations. Error ε between the real and estimated generator functions is represented in grey and the corresponding scale is in the right vertical axis. This error decreases as the number of subjects in the sample increases. The percentage error $\varepsilon_{p}$ was determined for cohorts of simulated data using (4) and is represented in black with the corresponding vertical axis in the left hand side. Similarly, the percentage error between the two functions also seems to decrease with the number of patients in the cohort.

We verified that the error between the real generating function and the estimated one, $\varepsilon$ given by (4), decreased as the number of subjects increased (Fig. 3). We tested progressively large samples of simulated data, ranging from a size of 100 up to 1,000 subjects, in a total of forty, ten for each of the four tested generator functions.

The error given by (5) is also represented in Fig. 3 and it can be modelled by a $\chi$^2^ distribution. In this case, it can also be observed that the error gets smaller as the number of subjects in the sample increases, meaning that the two distributions become more and more similar up to a point where the differences between them may be attributed to random aspects.

### Real data: the head and neck squamous cell carcinoma cohort

**Figure 4:**
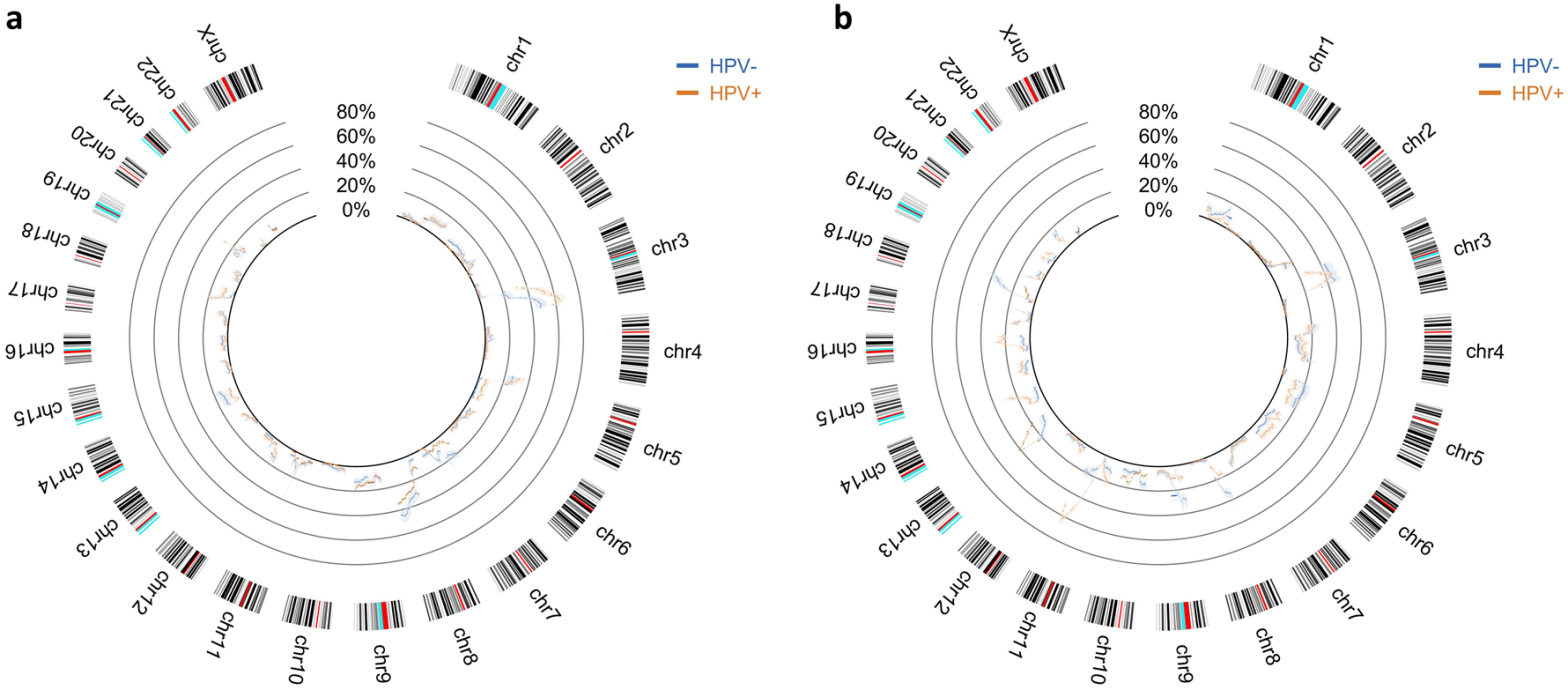
Estimated probability of alteration per chromosomic region, for two groups of patients in a Head and Neck Squamous Cell carcinoma cohort. In blue is represented the HPV-negative group and in orange the HPV-positive group. (**a**) Represents the gains in chromosomic material and (**b**) represents the losses of chromosomic material. Confidence bands are represented in the correspondent colour (shaded) around the probability distributions of each phenotypic group. Non-overlapping intervals of confidence can be observed that represent significant differences in probability of alteration between groups, meaning there is reason to believe the groups are different in those regions.

Since the data is real, we have no means to know what the real generator function could be for the probability of alteration so we had to assume that this probability is given by the estimated generator function derived from the data, $\hat{\Phi }\left( r \right)$. For each genomic region,$r$, a Bernoulli experience is assumed with probability $\hat{\Phi }\left( r \right)$ and a proportion confidence interval was estimated for each $\hat{\Phi }\left( r \right)$. It was assumed a Bernoulli experience because it is a random variable with only two possible outcomes, which can be normal or gain in one situation and normal or loss in the other case. One estimated generator function was determined for each phenotypic group (HPV-positive and HPV-negative) along with 95% confidence bands around the probability distributions. These functions were determined for gains and losses, separately, along the somatic chromosomes, excluding the sexual ones. These probability distribution profiles are represented in Fig. 4.

As it can be observed, even though for the majority of chromosomes the bands of confidence around both estimated generator functions overlap, there are cases in which this does not happen, which may be interpreted as existing statistical differences at the CNA level between the two groups, in those regions. This is especially evident in chromosomes 3, 8, 14 and 18 in the gain of genetic material image (Fig. 4a) and chromosomes 8, 11, 13 and 18 in the losses image (Fig. 4b). Another point that worth mention is the overall pattern of the alterations in the two groups that may be visually interpreted as a signature related to phenotype or clinical feature.

**Figure 5:**
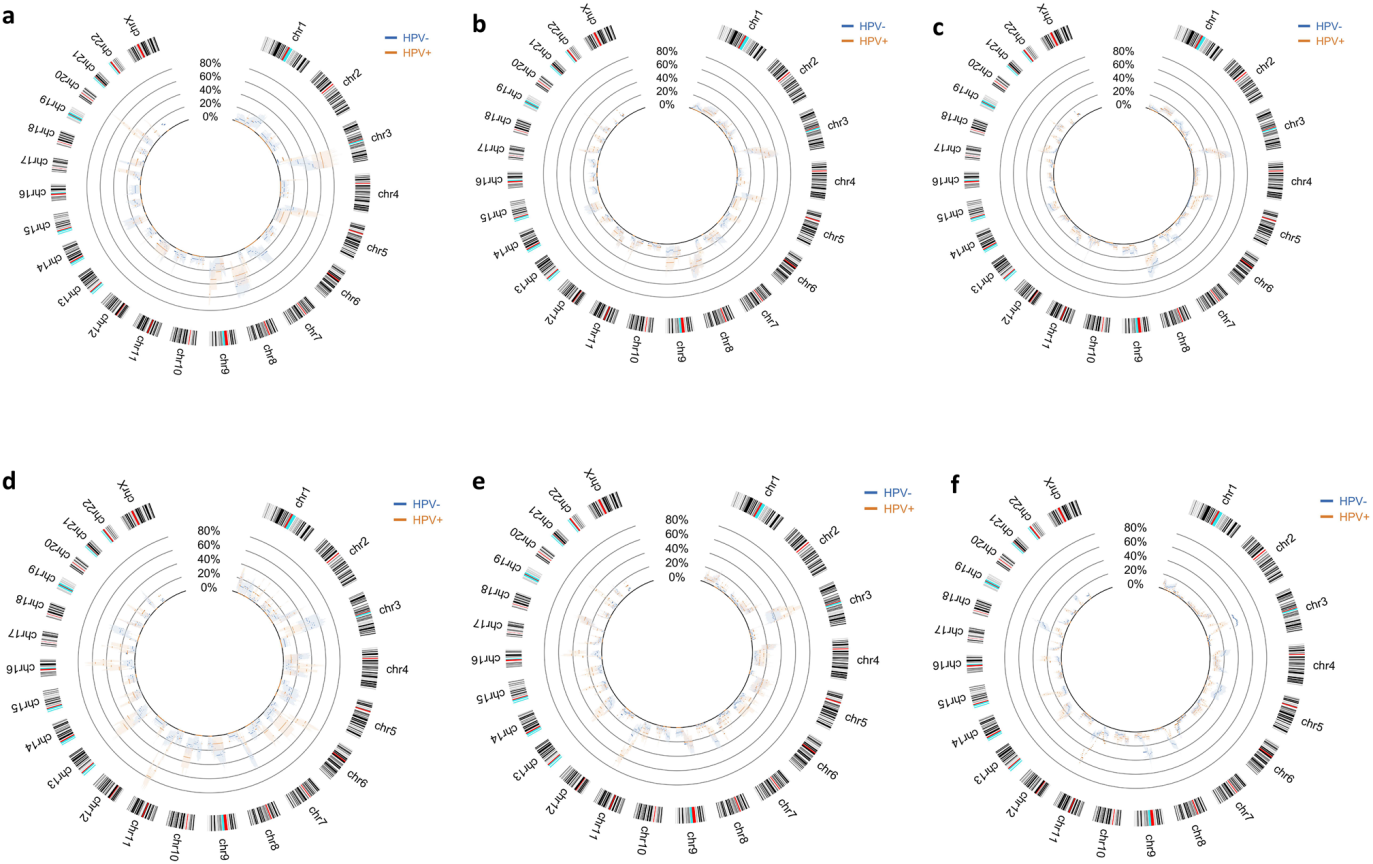
Estimated probability of alteration per chromosomic region, for two groups of patients in a Head and Neck Squamous Cell carcinoma cohort, in randomly drawn samples of different sizes. In blue is represented the HPV-negative group and in orange the HPV-positive group. (**a**) to (**c**) represent the gains in chromosomic material in samples of 50, 100 and 200 subjects; (**d**) to (**f**) represent the losses of chromosomic material in the corresponding samples of 50, 100 and 200 subjects. Confidence bands are represented in the correspondent colour (shaded) around the probability distributions of each phenotypic group. Non-overlapping intervals of confidence can be observed that represent significant differences in probability of alteration between groups.

Figure 5 portrays the genomic profiles obtained from three different sample sizes (50, 100 and 200 subjects). The profile varies with the sample size but the overall aspect is kept. Several samples of the same size were drawn and the results were similar (data not shown), which suggests that the process is robust. Additionally, confidence bands increase in amplitude as samples become smaller, which show that confidence is reduced in these cases, as expected. On contrary, larger sample sizes allow more confident comparisons to be drawn.

**Figure 6:**
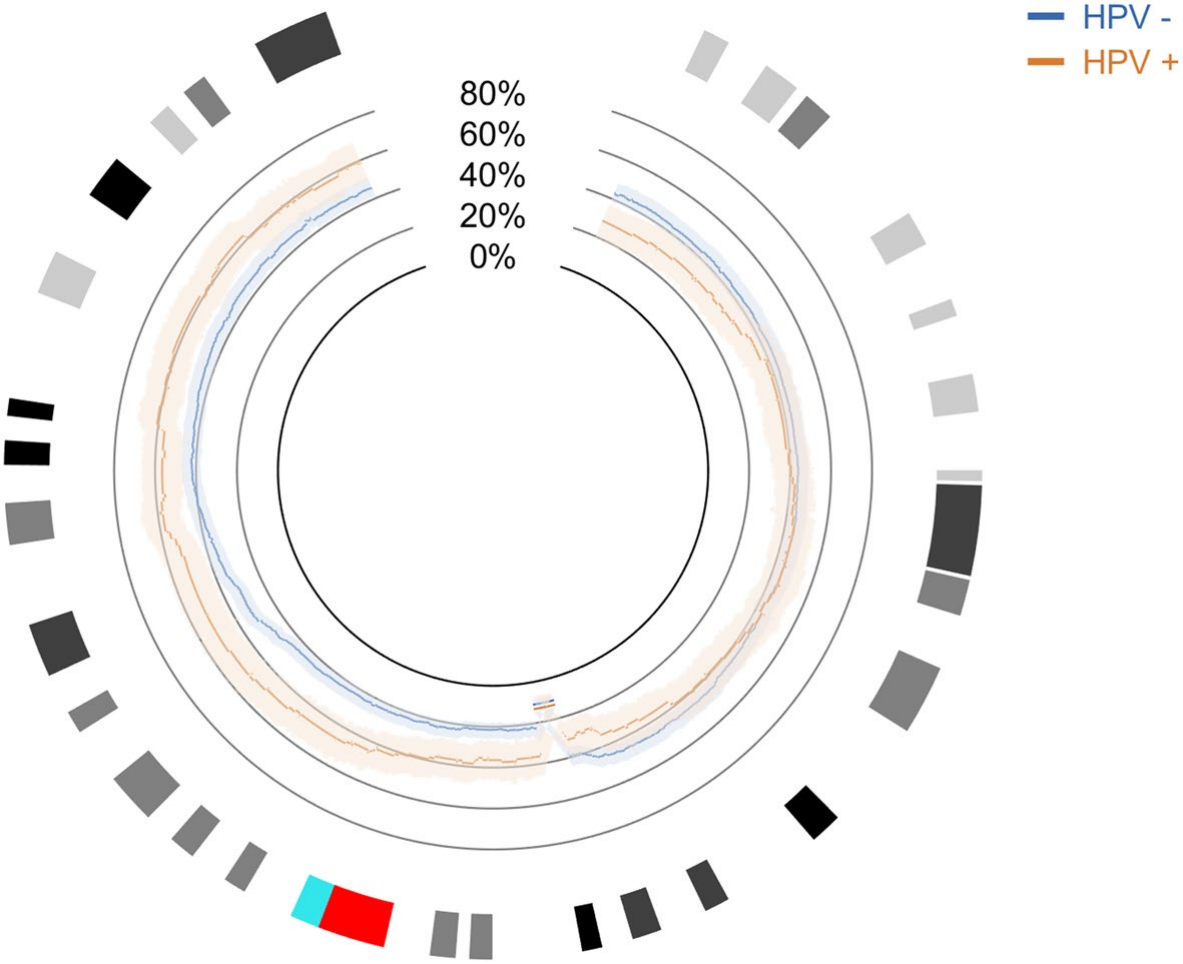
Estimated probability of alteration in chromosome 3, in a cohort of Head and Neck Squamous Cell carcinoma patients, for two groups (HPV-negative and HPV-positive) with confidence bands. In blue is represented the HPV-negative group and in orange the HPV-positive group. Gains and losses of chromosome material are shown together.

There is also an option to display a single chromosome, where probability distribution profiles for different groups can be added. Figure 6 represents probability distribution profiles for chromosome 3, as an example, in the HPV+ and HPV− groups, with confidence bands. Gains and losses are shown together.

**Figure 7:**
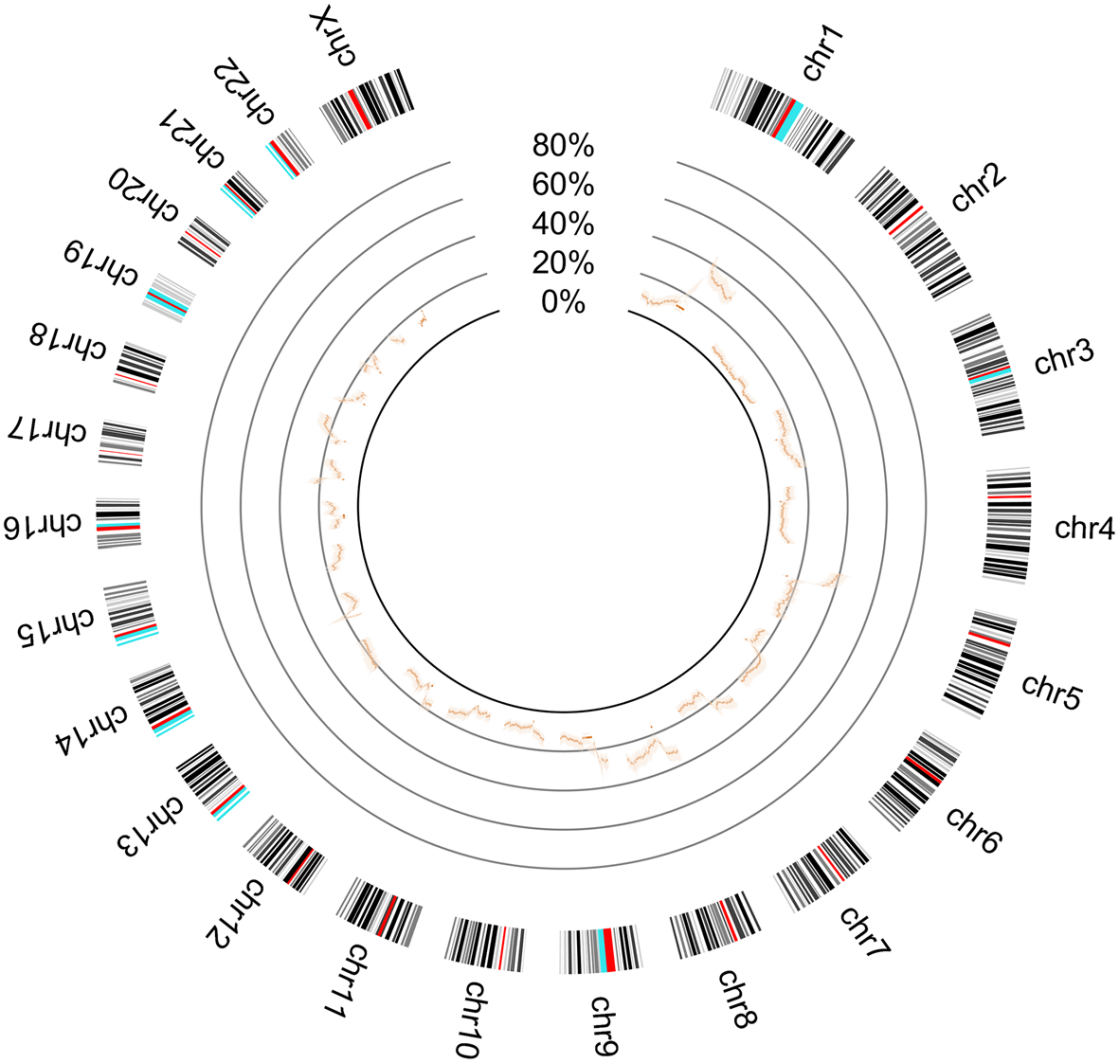
Determined probability of alteration per chromosomic region, in a cohort of lung adenocarcinoma, in all the somatic chromosomes. Shaded confidence bands are represented around the probability distribution.

A disease profile for lung adenocarcinoma was also established, based on the described methodology. This profile is represented on Fig. 7.

## Discussion

The study of CNAs is crucial to understand the development and progression of several conditions, the most prominent of those being cancer. Besides diseased states, DNA copy number is naturally variable and these alterations are commonly observed in normal populations. The presence of genomic fragile sites, that are more susceptible to breakage, play important roles in genomic instability. Common fragile sites are part of normal chromosome structure and are present in all individuals in a population. Under normal conditions, most common fragile sites are not prone to breakage^23–25^. However, the presence of fragile sites by itself seems not to be a deterministic indication that alterations should appear in the regions flanking or containing them, since not all fragile sites form breaks at the same frequency^25^. This fact seems to support that the phenomena of genomic copy number variability may be a stochastic process. In another work, we have proven that frequent breakpoints and their proximity to DNA repeat elements may play an important role in the genomic instability of HNSCC, however, we could not exclude the hypothesis that the presence of breakpoints contained or flanked by those repeat elements could be due to randomness^26^.

The method here described allows for a fast and efficient determination of potential target regions for cancer research. Other methods have been applied to solve this problem, those include CMAR, Significance Testing for Aberrant Copy numbers (STAC), Hierarchical Hidden Markov model (H-HMM), GISTIC, GISTIC 2.0, JISTIC and Kernel Convolution: A Statistical Method for Aberrant Region deTection (KC-SMART). Both GISTIC and JISTIC are very similar to GISTIC2.0, with JISTIC being an adaptation of GISTIC and GISTIC2.0 being the revised version of the former^15,27^. Some like CMAR and STAC require discretized copy number alteration states: loss, no aberration or gain; H-HMM uses three hidden states to model losses, absences of aberration and gains while GISTIC 2.0 requires segmented profiles. All methods used to determine recurrent regions aggregate all the sample profiles either in raw, segmented or discretized form, resulting in a significant reduction of passenger events in relation to recurring events^10,28^. GISTIC2.0 and KC-SMART take into account both the amplitude and frequency of the recurrent regions. GISTIC2.0 has the ability to detect focal recurring events enclosed in broader events, in a peel-off procedure that requires segmented data^10,28^. Not all of these approaches try to locate regions as it is done in this work, some methods focus on trying to locate common probes^10^. According to Rueda and Diaz-Uriarte many advantages come from finding common regions of alteration instead of probes, since the determination of regions allows for a compression of information, improves the association between CNAs and disease and facilitates the integration with other omics. Since genes are interrogated by multiple successive probes, there seems to be more relevant biological meaning behind finding recurring regions instead of a recurrent probe. KC-SMART is one of the methods that tries to find common probes instead of common regions of alteration and GISTIC also computes probe by probe statistics^10^. Methods, like MAR, CMAR and STAC also use data that have been discretized to the values “gain”, “loss” or “no change”, assuming that it has been done without error. This carries a possible loss of a great quantity of information, especially when aCGH is performed in very heterogeneous cell populations like in tumour cells^10^. GISTIC, smooths data per array, which makes difficult or even impossible comparisons between arrays^10^. Another drawback of GISTIC is the fact that is not designed to determine regions that are common to a small subset of samples, on the contrary, the method described here has the ability to detect those regions as well as regions of alteration common to a large group of subjects.

These methods tend to focus on the determination of single driver alterations or focal events, contrasting greatly with our proposed methodology, where probability distribution profiles of alteration are created from a pool of cancer patients’ genomic alterations. Although the lack of direct comparison with other methods should be considered as a limitation of the work, most published approaches may not be appropriate as a means of comparison with our method since the outputs are very different. Direct comparison between methods that have distinct outcomes would be rather complex and the conclusions about the performance of the method would be arguably biased.

Although the algorithm performed well in the determination of the common regions in the dataset used, it has also the same disadvantage of the other methods, which is the difficulty to compare results across cohorts. This may be caused by the fact that it may yield slightly different common regions of alteration depending on the samples included in the analysis as well as when another sample is added to a previously analysed cohort. This is a drawback of the method however, it should be noted that the probability distribution is not significantly affected and the main goal of efficiently compressing the data into common regions of alteration for a given set of samples is successfully achieved. In addition, this discrete function might be fitted by an analytical function (e.g. spline), which would allow comparisons between cohorts.

The goal of this work was to infer, from the sample, the probability distributions of disease profiles that characterize the population. In other words, to get the function that defines the disease’s genomic signature. This is a statistical process that has an associated confidence level, which can be translated into confidence bands around the probability distribution. The main advantage of inferring to the population is that comparison between cohorts is direct and conclusions can be drawn within a predetermined confidence level. The probability distributions, in this work, are called generator functions as they can be seen as the functions that generate each sample (cohort) from the same population presenting slightly different frequencies of alteration. Consequently, it is expected that cohorts with more subjects will present a distribution frequency closer to the generator function, which represents the real probability distribution of the population. The idea of using generator functions as the definition of a population is quite powerful since it will permit to distinguish between different phenotypic groups within the diseased population. Within the same disease, the method may also allow for the establishment of levels of severity between different groups, with the calculation of absolute distances between their probability distribution profiles. In this study, the simulated data used to test the partition algorithm was generated using regions with the same size for the sake of simplicity, however this is not what happens in the case of using real data. Another obstacle that arises from this interpretation of the problem is that the data must be clean from overlapping regions coming from the same tumours’ samples, otherwise there would be double counting biasing the resulting probability distribution. The method described does not take into account the amplitude of the signal (logarithmic ratios) or the discretized copy number values which, as described, may also be an advantage. Here, the common regions of alteration result from the overlapping of regions which are altered in different tumour profiles and, as such, there is, in the worst case scenario, negligible loss of information. The greatest advantage of this straightforward method is that it uses probability at its core and so the results are directly interpretable and easily inferable to the population and originate from the direct observation of real data.

It should be noted that a control group of normal, non-tumoral tissue was not considered for comparison with the real cancer data, since only a small number of copy number alterations was expected to be present in this tissue. Similarly, is not advisable to use tissue adjacent to the tumours as control, since many of the alterations present in the tumour may already be present in this tissue.

Aspects such as purity and ploidy were not studied in this work, since, the estimation of tumor purity and ploidy should be performed on the tumor sample of a single patient and when the absolute copy number is determined for a given segment and the copy number alteration is quantified it should already be known. In this work, we studied copy number alteration data available from The Cancer Genome Atlas, where the determination of the segments copy number had already been performed, presenting log2 segment means that are calculated relatively to the ploidy of the tumor sample of each subject.

Hypothetically, the purity of the samples could be determined by verifying the existence of outliers in the cohort, removing each patient at a time and comparing their profile of alteration with the one generated by considering the other patients together. However, by assuming that impurity has a lower probability of occurrence than purity and that, in the case of an outlier, is the impurity that explains the deviation from the sample’s profile when in fact many factors could influence that difference, makes it a poor measure.

Two different phenotypic groups based on HPV status were used to establish two disease profiles within the HNSCC cohort. Although the profiles are mostly undistinguishable, since the confidence bands around them overlap, there are differences that are clear. This result may be expected considering that, even though there is heterogeneity within the same tumour type, most alterations should be similar. However, due to the intervention of HPV on tumorigenesis, some alterations may become statistically different between both profiles. A profile for lung adenocarcinoma was also established to show the applicability of our methodology in a different cancer type. In this case, the differences between tumour types are quite marked as one can perceive by comparing Figs. 4 and 7. A distance measure between the tumour types could be computed from the function profiles, which would be highly informative.

The main objective of this work was, from microarray-based genome-wide technology data, to retrieve relevant information in the form of common altered regions in order to construct probabilistic genomic alteration profiles able to characterize the population in study. The approach we propose in this study was tested using simulated and real data. The simulation was used with the only purpose to validate the partition algorithm, which was accomplished. The probability distributions obtained from the studied tumour cohorts, show that we can establish differentiated profiles associated with different phenotypic characteristics, within a well-known confidence level. This approach can be a tool, not only for research purposes, but potentially for clinical applications either as a diagnostic tool, prognosis assessment or as a possible useful method for the establishment of adequate treatment and care plans.

## Data Availability

Source code is available at: https://github.com/LCGUC/Probability-distribution-of-CNAs Matlab Standalone available at: https://faculty.uc.pt/fcaramelo/en.
